# Stabilizing effects of geraniol on native and pathogenic M39R rhodopsin variants

**DOI:** 10.3389/fnins.2026.1799935

**Published:** 2026-03-20

**Authors:** Pol Fernandez-Gonzalez, Jenifar Das, Alejandro Cruz, Juan J. Perez, Pere Garriga

**Affiliations:** 1 Grup de Biotecnologia Molecular i Industrial, Centre de Biotecnologia Molecular, Departament d’Enginyeria Quimica, Universitat Politècnica de Catalunya-Barcelona Tech, Edifici Gaia, Terrassa, Catalonia, Spain; 2 Grup de Biotecnologia Molecular i Industrial, Centre de Biotecnologia Molecular, Departament d’Enginyeria Quimica, Universitat Politècnica de Catalunya-Barcelona Tech, ETSEIB, Barcelona, Catalonia, Spain

**Keywords:** protein misfolding, retinal degeneration, retinitis pigmentosa, rhodopsin, visual phototransduction

## Abstract

**Introduction:**

Mutations in rhodopsin are a major cause of autosomal dominant retinitis pigmentosa, frequently due to protein misfolding and reduced structural stability. The M39^1.34^R variant, located in transmembrane helix 1, is associated with sector retinitis pigmentosa and exhibits pronounced thermal and chemical destabilization.

**Methods:**

We investigated whether geraniol, a natural monoterpenoid alcohol with reported cytoprotective properties, can act as a pharmacological stabilizer of rhodopsin. Using purified pigments from bovine rod outer segments and recombinant wild-type and M39^1.34^R variants, we assessed photochemical integrity, thermal denaturation, hydroxylamine susceptibility, regeneration kinetics and Meta II fluorescence responses in the presence or absence of geraniol.

**Results:**

Geraniol did not interfere with chromophore binding, photobleaching, regeneration, or acid-induced Schiff base protonation. However, it significantly enhanced the thermal and chemical stability of rod outer segments-derived and recombinant wild-type rhodopsin. The M39^1.34^R mutant displayed severe thermal instability and increased susceptibility to hydroxylamine, but geraniol treatment nearly doubled its thermal half-life and improved chromophore retention, although without restoring wild-type stability levels. Remarkably, geraniol induced rapid and marked quenching of Trp265^6.48^ fluorescence in Meta II, indicating state-dependent interaction with the activated receptor.

**Discussion:**

These findings identify geraniol as a membrane-interacting small molecule capable of partially stabilizing structurally compromised rhodopsin variants. While incomplete, the rescue achieved supports the potential of monoterpenoid-based pharmacological chaperones for selected rhodopsin-linked retinal degenerations.

## 1 Introduction

G protein-coupled receptors (GPCRs) constitute the largest and most diverse family of membrane proteins in the human genome, with over 800 members being a part of it (Alexander et al., 2021; Pándy-Szekeres et al., 2023). GPCRs play pivotal roles in transducing extracellular signals into intracellular responses, thereby regulating a wide range of physiological processes (Weis and Kobilka, 2018). Notably, approximately one-third of all FDA-approved drugs target GPCRs, underscoring their significance in pharmacology and therapeutic development (Hauser et al., 2017; Sriram and Insel, 2018).

Among GPCRs, rhodopsin (Rho) stands out as a prototypical model, by being the first GPCR for which a high-resolution crystal structure was obtained (Palczewski et al., 2000). Rho is a light-sensitive receptor expressed in the rod photoreceptor cells of the retina, initiating the phototransduction cascade essential for vision under low light conditions (Hofmann et al., 2009; Lamb and Pugh, 2006). The elucidation of Rho’s structure has provided highly relevant insights into GPCR activation mechanisms, ligand binding and conformational dynamics (Zhou et al., 2012), serving as a foundational template for understanding GPCR function (Weis and Kobilka, 2018).

Mutations in the Rho gene are known to be a predominant cause of retinal degeneration diseases such as autosomal dominant retinitis pigmentosa (adRP) (Daiger et al., 2015; Ramon et al., 2014), a progressive disease characterized by the loss of photoreceptor cells leading to vision impairment and eventually to blindness (Verbakel et al., 2018). Today, over 150 pathogenic mutations in *RHO* have been identified (Athanasiou et al., 2018), many of which disrupt correct protein folding, stability and trafficking, ending in photoreceptor cell death (Krebs et al., 2010; Mendes et al., 2005).

The M39^1.34^R mutation in Rho [superscript refers to the general numbering system for GPCRs (Ballesteros and Weinstein, 1995)], located within the first transmembrane domain, has been associated with altered phototransduction kinetics (Toledo et al., 2011). Specifically, prior studies have reported a faster rate of transducin (Gt) activation for the M39^1.34^R mutant compared to wild type (WT) Rho, which has been interpreted as indicative of altered Meta II decay kinetics, though it should be noted that increased Gt activation rates may also arise from altered receptor-Gt affinity rather than exclusively from accelerated Meta II decay (Kawamura et al., 2013; Farrens, 2010). These functional alterations suggest that the M39^1.34^R mutant perturbs the normal activation and deactivation cycle of Rho, potentially contributing to photoreceptor dysfunction (Rakoczy et al., 2011).

Addressing the effects of misfolded Rho variants has led to the exploration of pharmacological chaperones, small molecules that can assist in the proper folding and stabilization of proteins (Tao and Conn, 2018; Chen et al., 2018). Geraniol, an acyclic monoterpenoid alcohol found in essential oils of various plants, has earned attention for its pharmacological properties, including anti-inflammatory and antioxidant effects (Chen and Viljoen, 2022; Maczka et al., 2020). Studies have suggested that geraniol can modulate endoplasmic reticulum stress responses and protect against cellular damage (Prasad and Muralidhara, 2014; Rekha and Selvakumar, 2014), indicating its potential role as a pharmacological chaperone. Additionally, some studies have suggested a potential interaction between geraniol and Rho (Becker et al., 2019; Kisselev et al., 1995).

Notably, structurally similar amphiphilic compounds such as octyl glucoside have been shown to bind opsin in a defined cavity, suggesting that small molecules with hydrophobic chains and polar headgroups can access specific binding sites on this receptor (Park et al., 2013).

In this study, we investigated the capacity of geraniol to act as a pharmacological chaperone for the M39^1.34^R Rho mutant. Here, we assess whether geraniol treatment can enhance the stability and proper folding of the M39^1.34^R variant, thereby ameliorating its functional deficits. Our findings aim to contribute to the development of targeted therapeutic strategies for RP with Rho gene mutations.

## 2 Materials and methods

### 2.1 Native Rho purification from bovine rod outer segments

All purification steps were performed at 4 °C under dim red illumination in a dark room to preserve pigment integrity. Native rod outer segments (ROS) were isolated from bovine retinas following a previously established protocol (Reyes-Alcaraz et al., 2011). ROS were solubilized by gentle incubation in PBS 1× buffer at pH 7.4, supplemented with 1% n-dodecyl-β-maltoside (DDM).

Following solubilization, samples were centrifuged at 1,500 g for 40 min at 4 °C to remove, and the supernatant containing the solubilized Rho was incubated with sepharose beads covalently linked to the monoclonal Rho-1D4 antibody (Cell Essentials, Boston, United States) for 3 h on a rotary shaker at 4 °C. After this, beads were washed three times with PBS 1× buffer containing 0.05% DDM and Rho was finally eluted by incubating with the same buffer supplemented with 100 μM of the 1D4-specific nonapeptide (TETSQVAPA), which completely displaces the pigment from the antibody.

The concentration of the purified Rho was determined by measuring its absorbance at 500 nm in the dark using a Cary 100Bio UV–Vis spectrophotometer (Varian). The instrument was equipped with water-jacketed cuvette holders connected to a circulating water bath to ensure precise temperature control, which was maintained by a Peltier accessory integrated with the spectrophotometer. All optical measurements were performed using a pigment concentration of approximately 0.5 μM, calculated using the appropriate molar extinction coefficient. All spectral recordings were performed between 250 and 650 nm with a bandwidth of 2 nm, a response time of 0.1 s, and a scan speed of 300 nm/min. Unless otherwise stated, the final concentration of Rho used in the experiments was approximately 0.5 μM.

### 2.2 Construction, expression and purification of bovine recombinant wild type Rho and M39^1.34^R mutant

The WT construct used in this study was previously available in the pMT4 plasmid vector. The M39^1.34^R Rho mutant was generated via site-directed mutagenesis using the QuikChange^®^ kit (Stratagene), following the manufacturer’s protocol. For protein expression, COS-1 cells were cultured to approximately 85% confluence in 15 cm culture dishes and transiently transfected with either WT or M39^1.34^R constructs using PEI, 80 μL of 1 mg/mL solution, and a DNA concentration of 30 μg per dish.

Twenty-four hours post-transfection, the culture medium was replaced with fresh DMEM to minimize PEI-induced cytotoxicity. After an additional 24 h, cells were collected and washed twice with 1× PBS (pH 7.4). Regeneration of the visual pigment was performed by incubating the cell pellets overnight at 4 °C under gentle agitation with 15 μM 11-*cis*-retinal (11CR) in 1× PBS (pH 7.4).

Pigment solubilization was achieved by incubating the cells at 4 °C in 1% DDM supplemented with 100 μM phenylmethylsulfonyl fluoride (PMSF), followed by ultracentrifugation at 35,000 rpm for 30 min at 4 °C. The solubilized pigments were subsequently immunopurified using Sepharose 4B resin covalently coupled to the monoclonal Rho-1D4 antibody. Incubation was carried out under continuous agitation at 4 °C for 3 h.

Elution of the bound pigment was performed using PBS 1× (pH 7.4) containing 0.05% DDM and 100 μM of the 1D4-specific nonapeptide (TETSQVAPA). The concentration of purified Rho was determined as described in the previous section.

### 2.3 Photobleaching and acidification of the purified pigments

The UV–Vis absorption spectra of purified samples were recorded in the dark over a wavelength range of 250–650 nm at a temperature of 20 °C. For photobleaching, samples were illuminated for 30 s using a Dolan Jenner FIBER-LITE MI-150 light source with a cut-off wavelength *λ* > 495 nm. Following light exposure, a second absorption spectrum was acquired to evaluate the spectral changes. After this, the solution was acidified by the addition of sulfuric acid to a final concentration of 40 mM, and a third spectrum was subsequently recorded.

Finally, to investigate the effect of geraniol on the physicochemical behavior of the pigments, the compound was added directly to the dark-adapted samples, prior to photobleaching. A final concentration of 50 μM geraniol was used in these assays.

The concentration of 50 μM was selected based on preliminary spectroscopic validation confirming absence of interference with rhodopsin measurements, and on the fact that geraniol is chemically stable under these conditions given its high boiling point and stability in aqueous solution at moderate temperatures.

### 2.4 Thermal stability of the pigments with and without geraniol

Thermal stability experiments were carried out at 48 °C, a temperature previously shown to provide an optimal balance between experimental resolution and the kinetic accessibility of thermal decay, particularly for thermally unstable Rho variants.

Spectroscopic measurements were conducted using recombinant WT and M39^1.34^R Rho pigments reconstituted with 11CR. For each condition, the baseline UV–Vis spectrum of the dark-adapted pigment was first recorded at 20 °C. In samples with geraniol treatment, the compound was added directly to the cuvette to a final concentration of 50 μM prior to thermal exposure.

Following compound addition and initial spectral acquisition, the cuvette was equilibrated to 48 °C, and absorbance spectra were recorded using a scan speed of 400 nm/min. A total of 70 scans were collected, one every 2 min, to monitor the thermal decay of the absorbance peak at 500 nm, which corresponds to the chromophore-bound state of Rho.

This procedure was performed for both untreated samples and samples containing 50 μM geraniol, allowing direct comparison of the thermal stability profiles under each condition. Data were fit to a single exponential decay function using Sigma Plot version 12.5 (Systat Software, Chicago).

### 2.5 Hydroxylamine reactivity of the pigments

Hydroxylamine hydrochloride was prepared at 1 M and adjusted to pH 7.0 prior to use. To evaluate the reactivity of Rho toward hydroxylamine, it was added to the dark-adapted pigment samples in 1× PBS containing 0.025% DDM to achieve a final concentration of 50 mM.

All measurements were carried out in the dark at 20 °C. Absorbance spectra were collected at 2-min intervals to monitor the progressive loss of the pigment visible absorption maximum and the concurrent appearance of a new absorption band at 365 nm, corresponding to the formation of retinal oxime.

Reaction kinetics were assessed by fitting the absorbance decay data to either a single-exponential decay model. Curve fitting and initial rate determination were performed using SigmaPlot software (version 12.5; Systat Software, Chicago).

### 2.6 Regeneration of Rho alone and with geraniol

To assess the effect of geraniol on Rho regeneration, photobleached samples were incubated with 11CR in the presence or absence of geraniol. Following complete photobleaching for 30 s, samples were incubated with 2.5-molar excess 11CR at 20 °C in the dark. For geraniol-treated samples, 50 μM geraniol was added simultaneously with 11CR.

Regeneration kinetics were monitored by recording absorbance spectra at regular time intervals (every 2 min with a scanning speed of 400 nm/min). The increase of the A_max_ absorption band was quantified and normalized to the initial dark-adapted spectrum. Regeneration rates were calculated by fitting the data to a single exponential recovery function using Sigma Plot version 12.5 (Systat Software, Chicago).

### 2.7 Meta II decay kinetics with geraniol addition

Meta II decay assays were carried out on a QuantaMaster 4 spectrofluorometer equipped with a TLC50 cuvette holder Peltier accessory for temperature control. Firstly, the W265^6.48^ fluorescence of dark-adapted samples was recorded at 20 °C until a steady baseline was reached. In dark-adapted samples, 11CR is tightly bound to the opsin with the β-ionone ring positioned close to the Trp residue, which quenches its intrinsic fluorescence. Right after illumination, retinal completely isomerizes to all-*trans*-retinal resulting in an increase of the fluorescence signal due to the fluorescence of W265^6.48^, which is no longer quenched.

Once the baseline was stable, samples were illuminated for 30 s using a Dolan Jenner FIBER-LITE MI-150 light source with a yellow cut-off filter (*λ* > 495 nm), and fluorescence was monitored until a plateau was reached, indicating maximal Meta II formation and stabilization. For collecting fluorescence spectra, samples were excited for 2 s at 295 nm with a 0.5 nm slit bandwidth, while blocking the excitation beam for 28 s by means of a shutter to avoid photobleaching of the samples. Trp emission was monitored at a wavelength of 330 nm with a slit bandwidth of 10 nm.

To evaluate the effect of geraniol on the stabilized Meta II state, 50 μM geraniol (final concentration) was added directly to the cuvette once the fluorescence plateau was achieved. The sample was briefly mixed by gentle pipetting, and fluorescence monitoring was continued using the same acquisition parameters.

Subsequently, hydroxylamine was added to a final concentration of 50 mM to assess the accessibility of the Schiff base and promote retinal release. The change in fluorescence upon hydroxylamine addition provides information about the effect of geraniol on Schiff base accessibility and Meta II conformation.

### 2.8 Molecular modeling

WT Rho structure was retrieved from the Protein Data Bank (PDB) (PDB code 1 U19) (Okada et al., 2004), while M39^1.34^R Rho was modeled from this same structure introducing the best rotamer of the corresponding mutation by the UCSF Chimera program (Pettersen et al., 2004).

With the aim of performing MD simulations reproducing physiological conditions, the protonation state of side chains was adjusted to pH = 7.5 using H++ web server (Gordon et al., 2005; Anandakrishnan et al., 2012), while geraniol protonation state was established by hand to ensure its correct description under such conditions.

Putative Rho allosteric cavities were determined by CavityPlus web server (Xu et al., 2018; Wang et al., 2023) employing the specific setting for shallow cavities (Cruz and Warshel, 2025), i.e., the parameters SEPARATE_MIN_DEPTH, MIN_ABSTRACT_LIMIT, SEPARATE_MAX_LIMIT, and MIN_ABSTRACT_DEPTH were set to values of 4, 187.5 Å^3^, 750 Å^3^, and 4, respectively.

To assemble WT and M39^1.34^R Rho binary complexes with geraniol, those cavities were filtered to determine the most prospective ones to host the considered compound, which was placed inside them by docking calculations. The GLIDE software (Friesner et al., 2004) was used to carry out such calculations, in which WT and M39^1.34^R Rho structures were used as receptors, receptor grids were generated from previous prospective cavities and 20 binding poses were calculated per grid that were ranked employing the empirical scoring function GlideScore (Friesner et al., 2006). The resulting structures of both Rho variants and their binary complexes with geraniol were subsequently embedded into a POPC membrane tapped with a 12 Å water layer containing Na^+^ and Cl^−^ ions to a concentration of 0.15 M at both sides, using the Packmol-Memgen package (Schott-Verdugo and Gohlke, 2019) within the AMBERTools22 package (Wang et al., 2004).

Prior to running the corresponding MD simulations, different systems were assembled using the tLeap module and the procedure recommended by the AMBER software package (Case et al., 2023). The force fields ff14SB (Maier et al., 2015) and Lipid21 (Dickson et al., 2022) were used for the protein and membrane atoms, respectively. Water molecules were described by TIP3P model (Jorgensen et al., 1983). Force field parameters for the lysine-retinal residue (LYR) were recovered from a previous work (Herrera-Hernández et al., 2022), while the specific parameters of geraniol were developed here employing the B3LYP/6-31G(d) level of theory to optimize its structure and the Merz–Kollman RESP procedure (Bayly et al., 2002) to assign atomic charges. The GAFF2 (Wang et al., 2004) library was used as the source for all these parameters. All performed MD simulations followed the same protocol, which consisted of 5 different steps: (1) minimization; (2) heating; (3) pressurization; (4) equilibration; (5) production. Initially, a mixed minimization that combines steepest descent and conjugate gradient methods of 40,000 minimization steps were employed to remove atomic clashes. After this, MD simulations were carried out using periodic boundary conditions. The system was gradually heated from 0 to 300 K at constant volume for a period of 400 ps applying harmonic restraints to the whole GPCR, and geraniol if present, with a force constant of 10.0 kcal mol^−1^ Å^−2^. Then, a pressurization process at 1 bar in the NTP ensemble was applied during 32 ns for adjusting system density to atmospheric pressure. Throughout this process, previous harmonic restraints were stepwise realized. Next, a 300 ns equilibration stage was performed at constant temperature (300 K) and pressure (1 bar) keeping the entire system free of restraints. Finally, a production period of 500 ns was calculated within the same isothermal-isobaric ensemble. It should be noted that the equilibration state for M39^1.34^R Rho binary complex with geraniol occupying cavity 3 (M39^1.34^R Rho:geraniol(3) complex) was enlarged 400 ns because the ligand partially left the cavity to reposition itself correctly.

As for general settings, the temperature was controlled by Langevin dynamics (Leach, 1996), while the pressure was adjusted by the Berendsen (Berendsen et al., 1984) barostat. A time step of 2 fs was used and all bonds and angles containing hydrogen atoms were restricted using the SHAKE (Ryckaert et al., 1977) algorithm. The nonbonding interactions have been calculated with a cutoff of 10 Å, while the long-range electrostatic interactions were handled by the particle-mesh Ewald (PME) procedure (Darden et al., 1993; Essmann et al., 1995).

The AMBER 22 GPU (CUDA) version of the PMEMD software package (Salomon-Ferrer et al., 2013; Le Grand et al., 2013) was employed to calculate steps 2–5, while step 1 was calculated using the corresponding CPU version of the SANDER software package (Pearlman et al., 1995) due to the higher numerical accuracy required. The analysis of the MD simulations was carried out with AmberTools22 (Case et al., 2023).

Finally, the Caver 3.0 program (Chovancova et al., 2012) was employed to identify prospective channels for hydroxylamine access to the orthosteric site. Apart from WT and M39^1.34^R Rho variants, chemical stability against hydroxylamine was only assessed for its corresponding binary complexes with geraniol occupying cavity 3 (WT Rho:geraniol(3) and M39^1.34^R Rho:geraniol(3) complexes), since this compound exclusively binds into this cavity. For each considered system, 50 evenly-spaced structures of its corresponding MD simulation were considered for that search. As for calculation of those channels, the carbon atom of the LYR imine group, which undergoes the hydroxylamine nucleophilic attack, was chosen as a starting point, while the default value was selected for the rest of the channel’s parameters, except for the probe radius that was set to a value of 0.75 Å for taking into account the small size and large flexibility of hydroxylamine. All visualizations and representations were performed using the VMD (Humphrey et al., 1996) and UCSF Chimera (Pettersen et al., 2004) programs.

### 2.9 Statistical analysis

All results are reported as mean ± SEM and were statistically evaluated using a two-tailed independent Student’s *t*-test, with statistical significance defined as *p* < 0.05.

## 3 Results

### 3.1 Spectroscopic validation of geraniol and initial characterization

**Figure 1:**
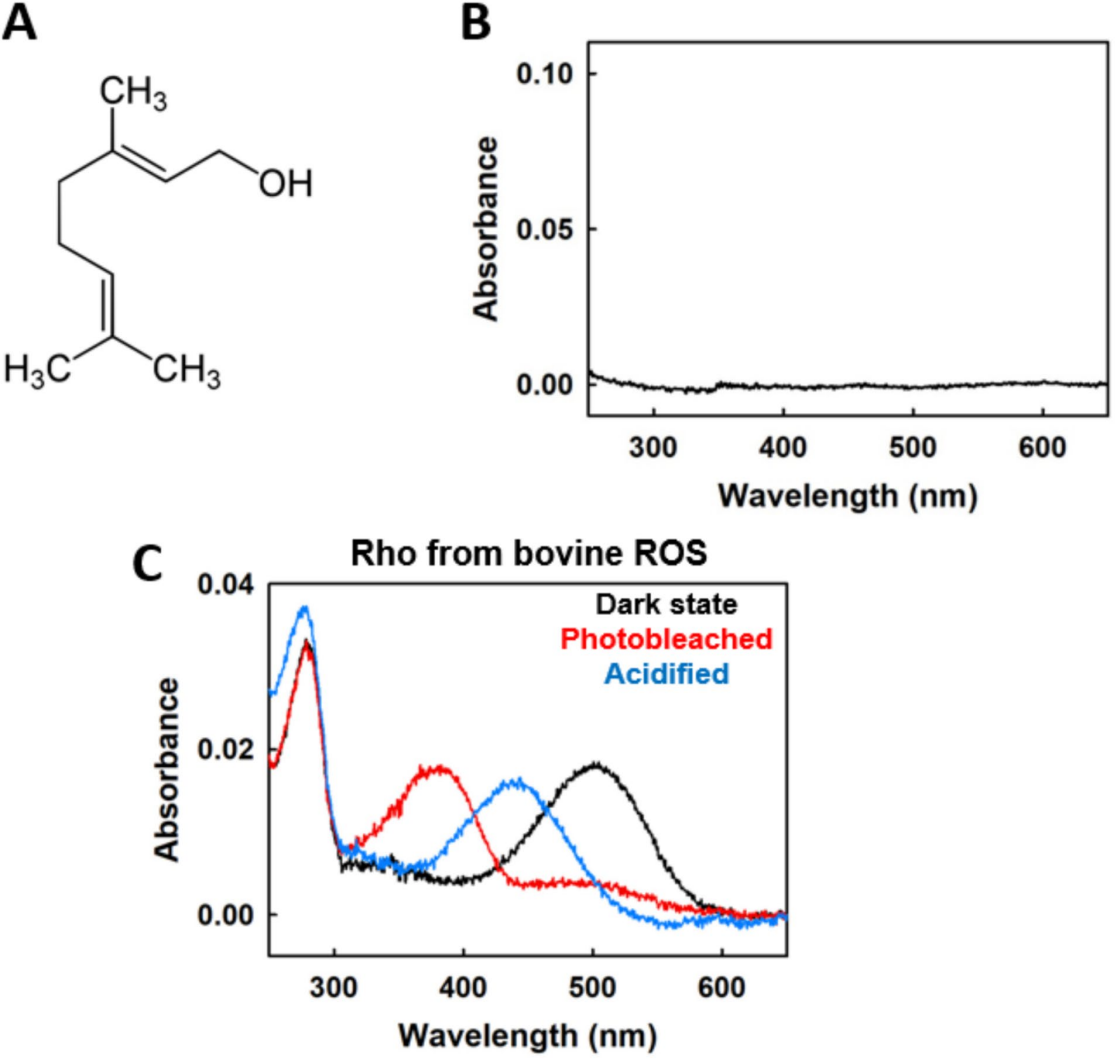
Molecular structure of geraniol and spectral properties in the presence of ROS-purified Rho. **(A)** Structural formula of the monoterpene alcohol geraniol. **(B)** Absorption profile of geraniol (50 μM) demonstrating absence of significant absorbance in the visible wavelength range. **(C)** Spectral characteristics of Rho isolated from ROS with 50 μM geraniol under different conditions: initial dark-adapted sample (black), following illumination with *λ* > 495 nm (red), and upon treatment with H_2_SO_4_ (blue). All spectroscopic measurements were conducted at 20 °C.

Prior to investigating the potential stabilizing effects of geraniol on Rho (Figure 1A), we first established that this monoterpene alcohol does not interfere with the spectroscopic methods employed throughout this study. UV–visible spectroscopy of 50 μM geraniol in PBS buffer revealed no detectable absorption in the visible region, confirming that geraniol would not contribute to measurements at Rho’s characteristic 500 nm absorption maximum or interfere with monitoring of photobleaching processes (Figure 1B).

To assess whether geraniol affects the fundamental photochemical properties of Rho, we performed standard photobleaching and acidification protocols on ROS-derived Rho in the presence of 50 μM geraniol. The dark-adapted pigment displayed the expected absorption maximum at 500 nm, characteristic of the 11CR chromophore bound to opsin (Park, 2014) (Figure 1C). Upon illumination with light >495 nm, the sample underwent complete photobleaching with loss of the 500 nm absorption and appearance of photoproduct absorption in the UV-blue region (Figure 1C). Subsequent acidification with sulfuric acid produced the characteristic spectral shift to ~440 nm, corresponding to the protonated Schiff base of denatured opsin (Ritter et al., 2004) (Figure 1C).

These spectral transitions were qualitatively indistinguishable from those observed in untreated Rho from bovine ROS (Wang et al., 2024), indicating that geraniol does not disrupt chromophore binding, prevent light-induced isomerization, or interfere with the conformational changes associated with photoactivation and chemical denaturation. This spectroscopic characterization validates the use of 50 μM geraniol as a working concentration for all subsequent stability and functional assays.

### 3.2 Thermal stability of Rho derived from ROS with geraniol

To quantitatively evaluate whether geraniol affects Rho conformational stability, we performed thermal denaturation experiments at 48 °C using ROS-derived pigment. This temperature was selected to accelerate protein unfolding while maintaining experimentally tractable denaturation kinetics.

**Figure 2:**
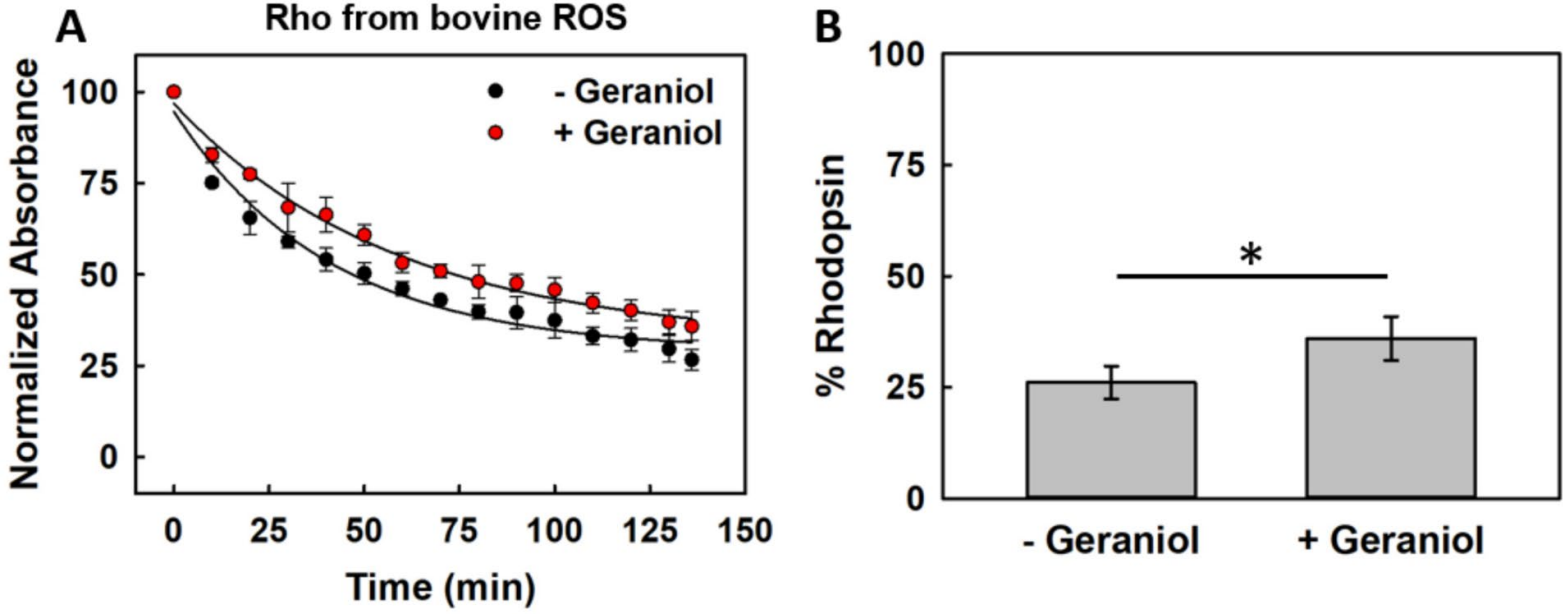
Thermal stability of ROS-derived Rho at 48 °C with and without geraniol treatment. **(A)** Time-dependent loss of visible absorbance maximum during thermal challenge in control conditions (black) versus samples containing 50 μM geraniol (red). **(B)** Percentage of intact chromophore-bound protein remaining after 140-min incubation at elevated temperature. Values shown as means ± SEM from three independent experiments (*n* = 3, *p* < 0.05).

Thermal stress induced progressive loss of the 500 nm absorption in both untreated and geraniol-treated samples, consistent with chromophore release and protein denaturation (Figure 2A). However, distinct differences emerged in the decay kinetics between conditions. Throughout the 140-min incubation period, samples containing 50 μM geraniol consistently maintained higher absorption levels compared to untreated controls.

Quantitative analysis of pigment retention after complete thermal treatment revealed that untreated samples preserved only 26.0 ± 3.7% of their initial absorption, whereas geraniol-treated samples retained 35.9 ± 4.8% (Figure 2B). This difference represents a statistically significant increase in protein stability. The protective effect was reported throughout the time course, with geraniol-treated samples showing consistently slower denaturation rates.

These results demonstrate that geraniol provides measurable protection against heat-induced Rho denaturation. The magnitude of stabilization, while modest in absolute terms, represents an improvement in protein resistance to thermal stress that could be physiologically relevant for marginally stable mutant variants.

### 3.3 Chemical stability assessment of native Rho with geraniol

In order to determine whether geraniol affects the accessibility of the chromophore binding site, we evaluated the susceptibility of ROS-derived Rho to hydroxylamine-mediated Schiff base hydrolysis (Liu et al., 2013). Samples were incubated with 50 mM hydroxylamine at 20 °C for 140 min in the presence or absence of 50 μM geraniol.

**Figure 3:**
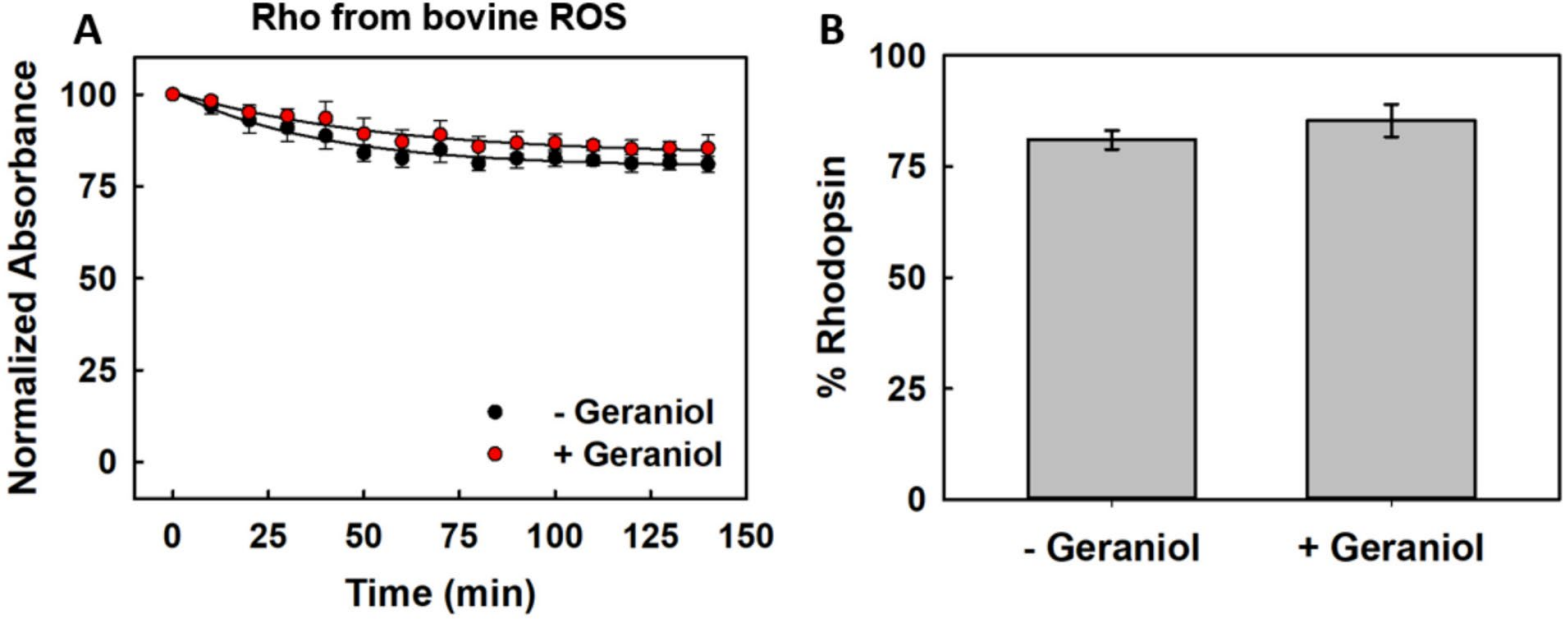
Chemical stability of ROS-derived Rho against nucleophilic attack in the presence of geraniol at 20 °C. **(A)** Time-dependent reduction in peak visible absorbance upon treatment with 50 mM hydroxylamine for control Rho (black) versus Rho with 50 μM geraniol (red). **(B)** Quantitative analysis of preserved dark-state protein following 140 min of hydroxylamine exposure. Error bars denote standard error across triplicate experiments (*n* = 3).

Both untreated and geraniol-treated samples exhibited high resistance to hydroxylamine attack, with only gradual decreases in the 500 nm absorption over the incubation period (Figure 3A). The decay profiles showed subtle kinetic differences, with geraniol-treated samples displaying slower rates of chromophore loss compared to untreated controls.

Quantitative analysis after 140 min revealed that untreated samples retained 81.0 ± 2.2% of their initial absorption, while geraniol-treated samples preserved 85.4 ± 3.6% (Figure 3B).

The high baseline stability of native Rho against hydroxylamine, retaining over 80% of chromophore even without treatment, limited the dynamic range for detecting protective effects. Nevertheless, the trend toward enhanced chemical stability in the presence of geraniol is consistent with the thermal stability data, suggesting that geraniol provides general stabilization of the protein structure rather than specifically protecting the chromophore binding pocket.

### 3.4 Chromophore regeneration kinetics of native Rho with geraniol

To assess whether geraniol influences the ability of photobleached opsin to rebind chromophore and reform functional pigment, we monitored regeneration kinetics following addition of exogenous 11CR to illuminated ROS samples.

**Figure 4:**
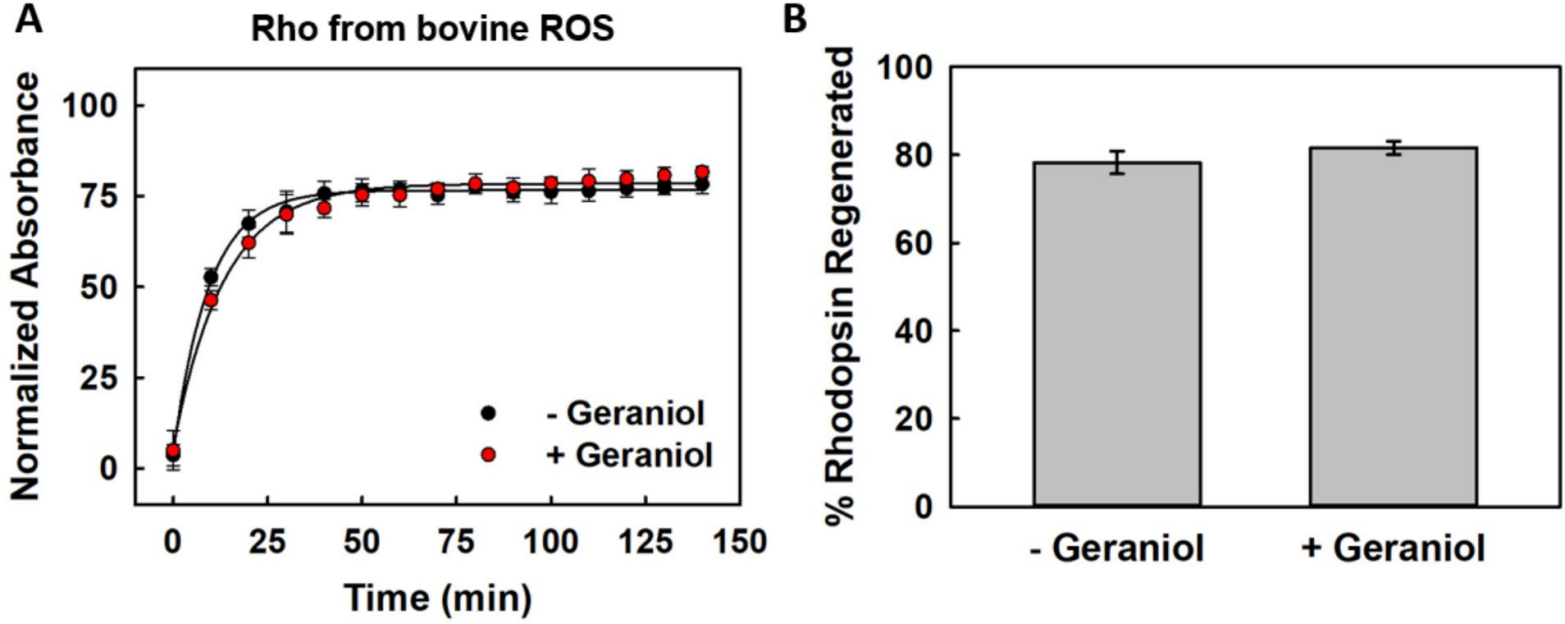
Chromophore regeneration of ROS-derives Rho using exogenous 11CR. **(A)** Kinetic profiles of visible *λ*_max_ recovery after 11CR addition to light-bleached Rho samples without (black) or with (red) 50 μM geraniol at 20 °C. **(B)** Extent of pigment reformation measured at 140 min post-addition. Values shown as means ± SEM from three independent experiments (*n* = 3).

The recovery of the 500 nm absorption band was monitored over 140 min (Figure 4A). Both conditions showed rapid initial regeneration during the first 30 min, followed by a gradual approach to plateau. The regeneration profiles were virtually superimposable throughout the entire time course, with no detectable differences in either the rate or extent of chromophore rebinding.

Quantitative analysis of final regeneration levels confirmed this observation (Figure 4B). Untreated samples achieved 78.5 ± 2.9% regeneration, while geraniol-treated samples reached 81.6 ± 1.4% of the initial dark-adapted absorption. This minor difference falls within experimental variation and does not represent a statistically significant effect.

The preservation of normal regeneration kinetics demonstrates that geraniol does not sterically obstruct the chromophore binding pocket or alter the conformational flexibility required for 11CR accommodation. Both the rate and extent of pigment reformation remained unchanged, indicating that geraniol’s stabilizing effects observed during thermal stress do not translate into altered chromophore binding dynamics.

### 3.5 Meta II decay and geraniol-induced fluorescence quenching

Intrinsic Trp fluorescence was employed to examine potential interactions between geraniol and the photoactivated receptor. During Meta II formation in ROS-derived Rho, Trp265^6.48^ fluorescence increases as the β-ionone ring of retinal separates from its quenching position in the dark state (Schafer et al., 2020).

**Figure 5:**
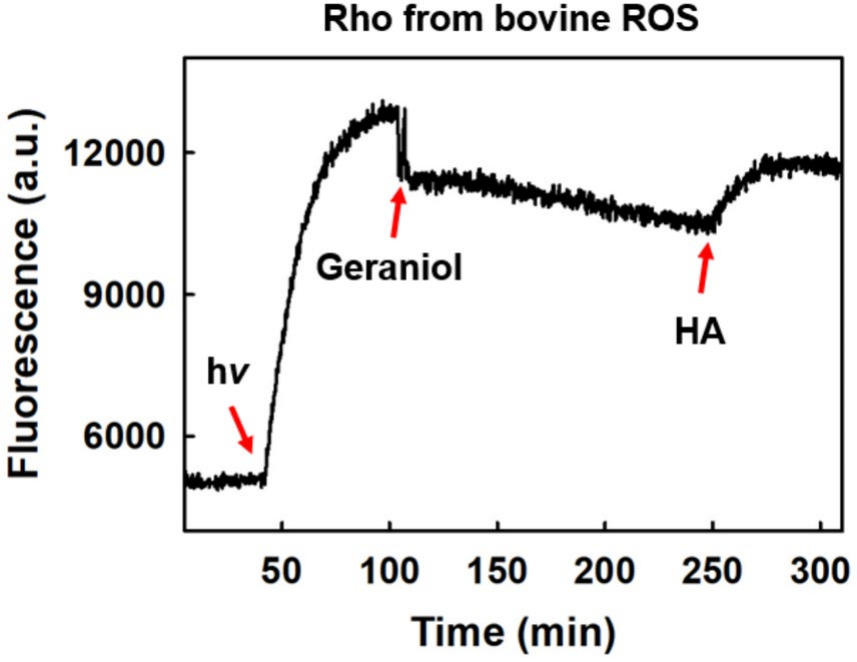
Tryptophan fluorescence changes in Meta II rhodopsin following geraniol and hydroxylamine treatment. Light-activated rhodopsin displaying enhanced Trp265^6.48^ fluorescence characteristic of Meta II formation was exposed to 50 μM geraniol, resulting in marked fluorescence suppression. Addition of hydroxylamine (HA) led to partial signal recovery. All measurements conducted at 20 °C.

Following illumination, samples reached a fluorescence plateau indicating maximal Meta II formation (Figure 5). Addition of 50 μM geraniol at this point induced an immediate and pronounced decrease in fluorescence intensity. This initial rapid quenching was followed by a slower, gradual decline in fluorescence that continued over the subsequent monitoring period, suggesting either ongoing conformational adjustments in the Meta II-geraniol complex or, alternatively, the gradual formation of subsequent photointermediates such as Meta III, which are known to occur over extended timescales and could contribute to the observed fluorescence changes. Subsequent addition of 50 mM hydroxylamine partially restored fluorescence. This recovery may indicate that geraniol’s quenching effect depends on the intact Meta II conformation rather than representing irreversible Trp modification.

The biphasic nature of the response, with an immediate pronounced quenching followed by a slower gradual decline, suggests that at least two processes may be operating. The rapid component is consistent with a direct effect of geraniol on the Meta II state, potentially through binding in proximity to Trp265^6.48^ or induction of local conformational changes affecting the Trp microenvironment, though direct evidence for specific binding interactions at this site is not available from the present data. The slower component may reflect Meta III formation or other late photointermediate transitions.

### 3.6 Photochemical properties of WT and M39^1.34^R in the presence of geraniol

Rho was purified from transfected COS-1 cells to evaluate the photochemical properties of both WT and M39^1.34^R variants. Both proteins were reconstituted with 11CR and subjected to sequential photobleaching and acidification protocols in the presence of 50 μM geraniol.

**Figure 6:**
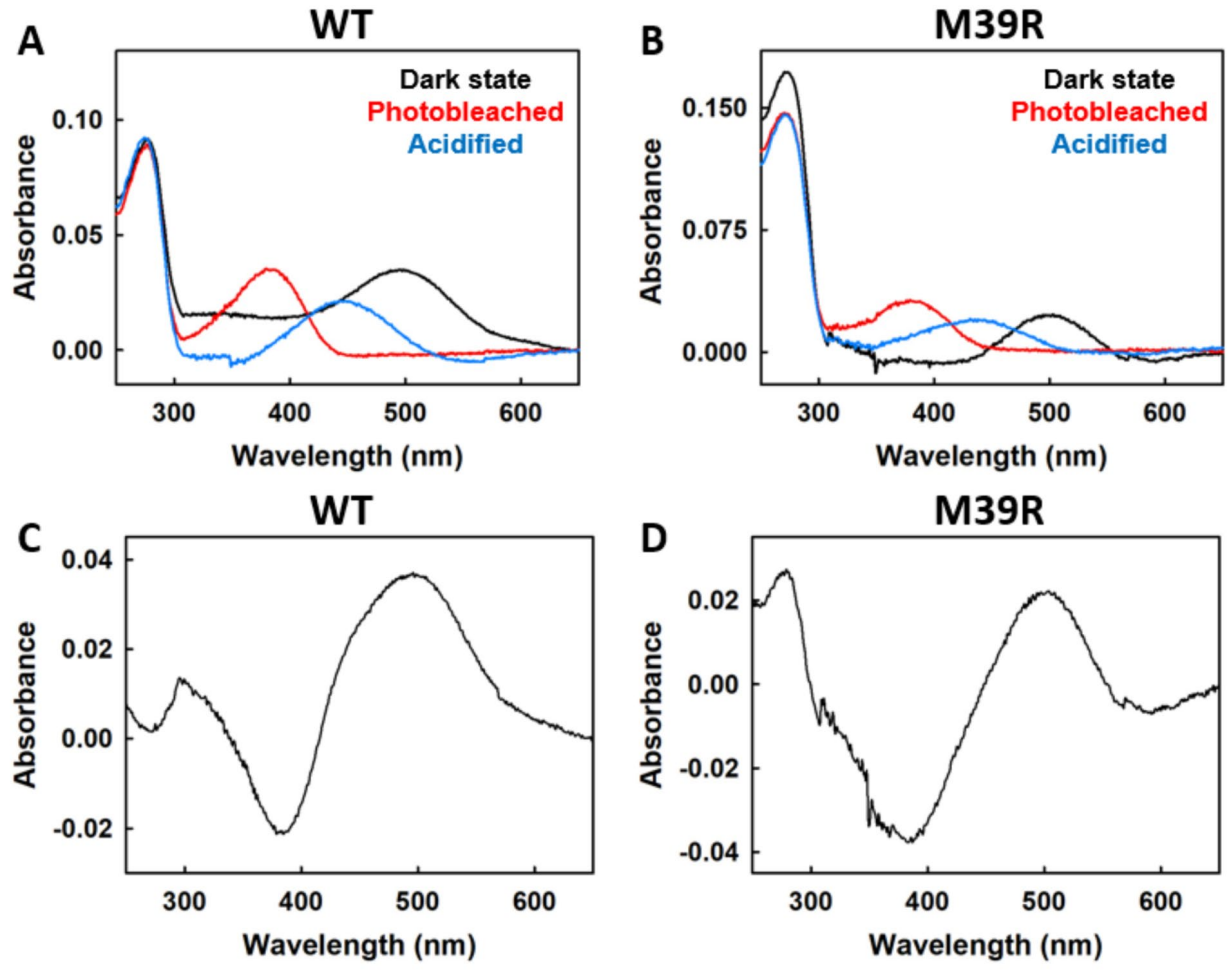
Spectroscopic analysis of WT and M39^1.34^R Rho photobleaching with geraniol treatment. Absorption profiles of WT **(A)** and M39^1.34^R mutant **(B)** Rho in 50 μM geraniol: native dark state (black), following illumination at *λ* > 495 nm (red), and upon acid denaturation with H_2_SO_4_ (blue). Differential spectra obtained by subtracting bleached from dark spectra for WT **(C)** and M39^1.34^R **(D)**, demonstrating typical photolysis patterns. All spectroscopic data collected at 20 °C.

WT Rho exhibited the expected spectroscopic behavior (Figure 6A). The dark-adapted pigment displayed an absorption maximum at 500 nm, which was completely abolished upon illumination with light >495 nm, accompanied by the appearance of photoproduct absorption in the UV-blue region. Subsequent acidification with sulfuric acid produced the characteristic shift to ~440 nm, corresponding to the protonated Schiff base.

M39^1.34^R demonstrated qualitatively similar photochemical properties (Figure 6B). The mutant formed a photoactive pigment with absorption at 500 nm in the dark state, underwent normal photobleaching upon illumination, and showed the expected acidification response. Difference spectra (dark minus bleached) for both variants confirmed proper photoisomerization, displaying the characteristic positive peak at 500 nm and negative peak in the UV-blue region (Figures 6C,D).

These results establish that the M39^1.34^R mutation does not abolish chromophore binding or prevent the conformational changes associated with photoactivation. The preservation of normal photochemical behavior in the presence of geraniol further confirms that the compound does not interfere with fundamental light-sensing mechanisms. The ability of M39^1.34^R to form photoactive pigment indicates that the mutation primarily affects protein stability rather than chromophore binding capacity.

### 3.7 Thermal stability of WT and M39^1.34^R in the presence of geraniol

The impact of the M39^1.34^R mutation on protein stability and geraniol’s protective capacity was quantitatively assessed through thermal denaturation experiments at 48 °C for both recombinant variants.

**Figure 7:**
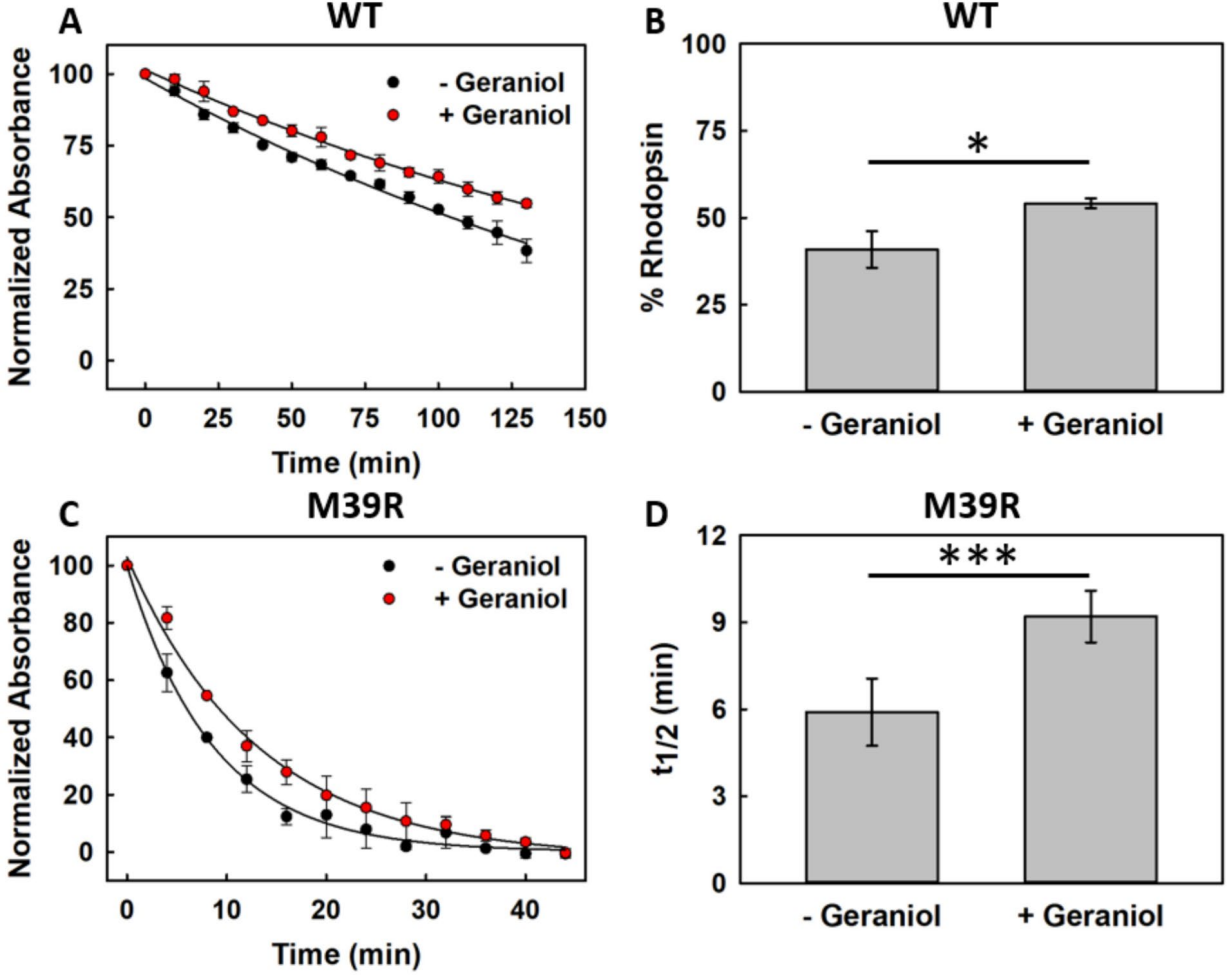
Thermal stability assay of recombinant WT and M39^1.34^R Rho at 48 °C. **(A)** Time-dependent stability profiles of WT Rho without (black) or with (red) 50 μM geraniol supplementation. **(B)** Quantification of intact WT protein remaining after 140 min thermal stress (*n* = 3, *p* < 0.05). **(C)** Kinetics of M39^1.34^R degradation demonstrating complete loss of chromophore within 40 min. **(D)** Calculated *t*_1/2_ values for M39^1.34^R thermal inactivation (*n* = 3, *p* < 0.001).

WT Rho underwent progressive thermal denaturation over 140 min, with similar decay profiles for untreated and geraniol-treated samples (Figure 7A). Quantitative analysis revealed that untreated WT retained 41.2 ± 4.6% of initial absorption, while geraniol-treated samples preserved 53.9 ± 1.8% (Figure 7B). This increase in pigment retention represents a statistically significant enhancement of thermal stability.

M39^1.34^R exhibited drastically compromised thermal stability compared to WT (Figure 7C). The mutant underwent rapid and complete denaturation, with total loss of absorption within 40 min under both conditions. Although both untreated and geraniol-treated M39^1.34^R samples showed stability loss, subtle differences emerged in their decay kinetics. Half-life analysis quantified this effect, revealing that untreated M39^1.34^R displayed a t_1/2_ of 5.9 ± 1.2 min, while geraniol-treated samples showed a t_1/2_ of 9.2 ± 0.9 min (Figure 7D). This increase in half-life was statistically significant.

The thermal instability of M39^1.34^R, losing all chromophore within 40 min compared to WT retaining ~50% after 140 min, demonstrates the destabilizing effect of this mutation. While geraniol provided some protection to the mutant, almost doubling its half-life represents only partial rescue, with absolute stability remaining far below WT levels even with treatment.

### 3.8 Chemical stability of WT and M39^1.34^R in the presence of geraniol

Hydroxylamine reactivity experiments were performed on both recombinant variants to evaluate whether geraniol’s protective effects extend to chemical perturbation and whether the M39^1.34^R mutation affects Schiff base accessibility since hydroxylamine selectively cleaves the linkage between 11CR and Lys296^7.43^ (Liu et al., 2013).

**Figure 8:**
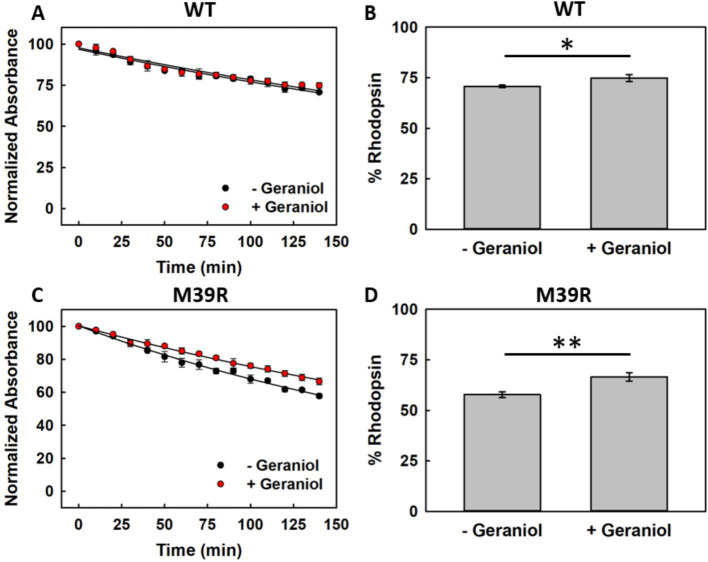
Hydroxylamine-mediated degradation of recombinant WT and M39^1.34^R Rho. **(A)** Time-dependent WT rhodopsin destabilization by 50 mM hydroxylamine at 20 °C without (black) or with (red) 50 μM geraniol. **(B)** Quantification of remaining intact WT protein following 140-min hydroxylamine exposure (*n* = 3, *p* < 0.05). **(C)** Kinetic profile of M39^1.34^R susceptibility to hydroxylamine attack. **(D)** Fraction of M39^1.34^R pigment preserved after 140-min treatment (*n* = 3, *p* < 0.01).

WT Rho showed gradual absorption decay over 140 min when incubated with 50 mM hydroxylamine at 20 °C (Figure 8A). Minimal differences were observed between untreated and geraniol-treated samples throughout the time course. Quantitative analysis revealed that untreated WT retained 70.7 ± 0.6% of initial absorption, while geraniol-treated samples preserved 74.8 ± 1.6% (Figure 8B). This modest improvement achieved statistical significance.

On the other hand, M39^1.34^R demonstrated reduced chemical stability compared to WT, with more pronounced decay under hydroxylamine treatment (Figure 8C). Final retention analysis showed that untreated M39^1.34^R preserved only 57.8 ± 1.4% of initial absorption, while geraniol-treated samples retained 66.6 ± 2.0% (Figure 8D). This improvement in chromophore retention was statistically significant and proportionally larger than the protection observed for WT.

The enhanced susceptibility of M39^1.34^R to hydroxylamine attack, indicates increased Schiff base accessibility in the mutant. Geraniol provided greater relative protection to M39^1.34^R than to WT, suggesting that the compound’s stabilizing influence becomes more apparent when protein structure is compromised. Nevertheless, even with geraniol treatment, M39^1.34^R chemical stability remained substantially below WT levels.

### 3.9 Molecular modeling

#### 3.9.1 Local differences around mutated position

**Figure 9:**
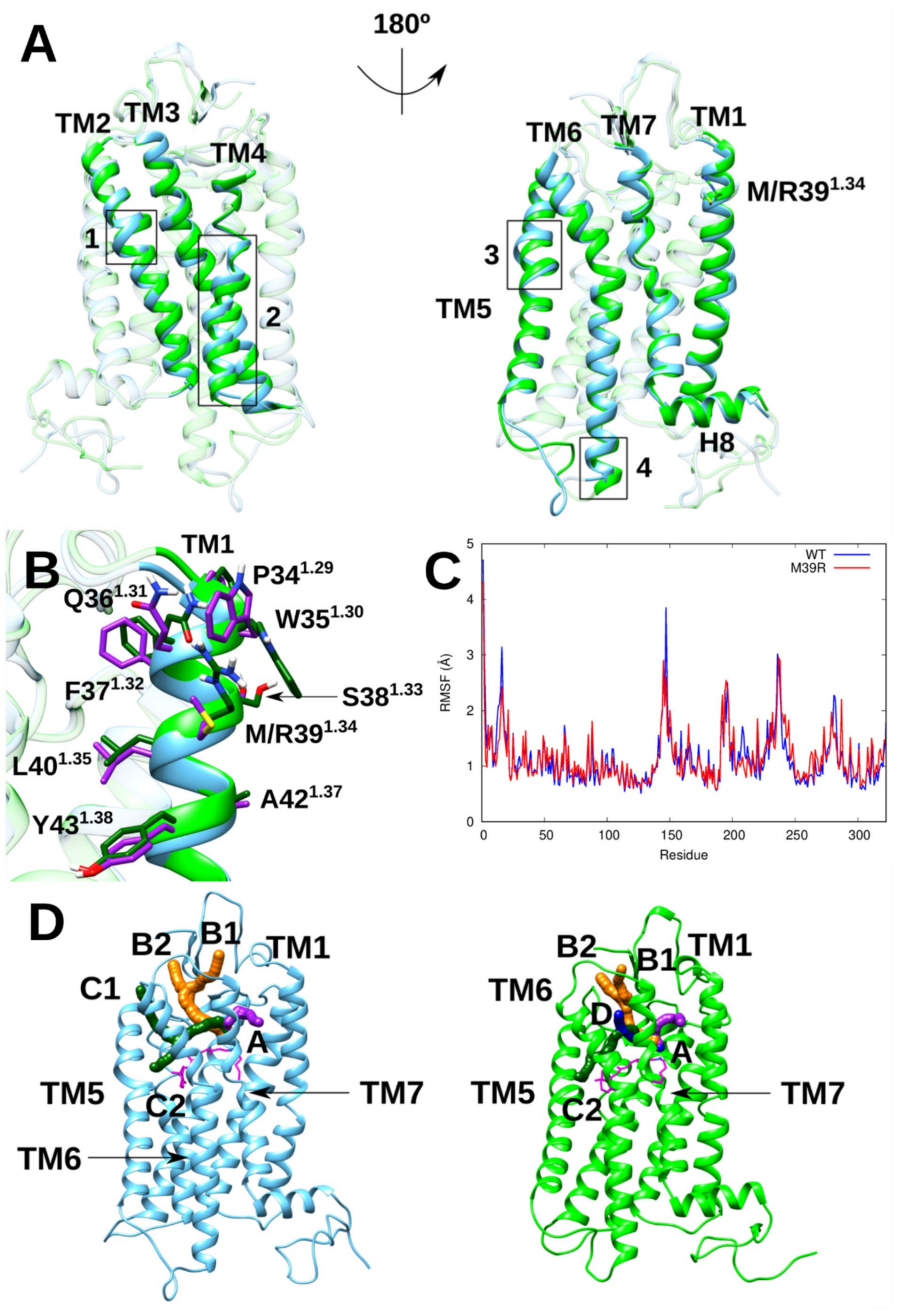
Main differences between WT and M39^1.34^R Rho. **(A)** Structural overlap of WT (cyan) and M39^1.34^R (green) Rho representative structures highlighting their main structural differences induced by the considered mutation: (1) Correction of structural defects present on TM2; (2) TM4 helical content loss at the membrane region and clockwise rotation of the helical structure encompassed from the membrane region to its intracellular end (considering the extracellular side as a reference system); (3) Correction of structural defects present on TM5 (4); Helical folding of ICL3 region prior to TM6. **(B)** Environment around the mutated position 1.34 for WT and M39^1.34^R Rho (secondary structure in cyan and green, while side chains in purple and dark green, respectively). In case of M39^1.34^R Rho, simultaneous interaction between Trp35^1.30^, Gln36^1.31^, and Arg39^1.34^ causes a stress point that ends up destabilizing its transmembrane bundle. **(C)** Per-residue RMSF for WT and M39^1.34^R Rho. For the sake of clarity, the N-terminus segment subsequent to H8 has not been depicted due to its substantial thermal fluctuations in both Rho variants. **(D)** Channels that connect the LYR imine group with the GPCR outer region for WT and M39^1.34^R Rho (in cyan and green, respectively). These two Rho variants exhibit 3 common channels (A, B, and C2), while channels C1 and D are characteristics of WT and M39^1.34^R Rho, respectively. For M39^1.34^R Rho, its mutant Arg plays a key role attracting hydroxylamine molecules.

As seen before, M39^1.34^R is located at TM1 extracellular end in its position 1.34 and supposes the introduction of an amino acid whose nature is totally different in comparison to the original one (the original hydrophobic Met is replaced by a positively-charged Arg). The main difference observed at that region during the molecular dynamics (MD) simulations of both Rho variants is that the polar side chains of Trp35^1.30^ and Gln36^1.31^ reorganize to interact with Arg39^1.34^ for M39^1.34^R Rho. In this case, Trp35^1.30^ establishes a cation-*π* interaction with Arg39^1.34^, while Gln36^1.31^ forms a hydrogen bond via its side chain keto group with one of the Arg39 side chain NH_2_ groups (Figure 9B). Such interactions are not possible in WT Rho due to the absence of the mutant Arg. Despite the energetically favorable nature of this simultaneous interaction within the triad and the resulting stabilization of its surrounding region, this causes a stress point at that region for M39^1.34^R Rho, constraining its conformational freedom and modifying its structural dynamics, which, in turn, induces wider thermal fluctuations on the remaining GPCR (see RMSF analysis for more details). It should be pointed out that alternative interpretations are possible., although according to present calculations, the aforementioned rationalization is the most plausible.

#### 3.9.2 Structural comparison and thermal stability of WT and M39^1.34^R Rho

Clustering analysis revealed that both WT and M39^1.34^R Rho maintain similar overall conformations during MD simulations, with all structures gathering within a single cluster using a 1.5 Å RMSD threshold for helix α backbone atoms. Unlike other RP-associated mutations, M39^1.34^R does not severely disrupt Rho structure. The primary structural changes include: partial loss of helical content in TM4 at the membrane region accompanied by a clockwise rotation of the helix from membrane region to intracellular terminus; correction of structural defects present in TM2 and TM5 of WT Rho; and adoption of helical structure by the ICL3 region preceding TM6 (Figure 9A).

Secondary structure analysis confirmed highly similar content between variants (Supplementary Table S1), though per-helix breakdown revealed specific changes (Supplementary Tables S2, S3). Notably, TM4 showed reduced 3–10 helix content at the membrane region, while TM2 and TM5 exhibited slightly increased alpha-helical content corresponding to defect correction.

RMSF analysis indicated reduced thermal stability for M39^1.34^R Rho compared to WT (1.324 Å vs. 1.223 Å), with systematically higher fluctuations across most regions (Figure 9C and Supplementary Table S4). Specific regions showed notable deviations: residues 12–16 (N-terminus), 147–148 (ICL2), 231–236 (ICL3), and 281–283 (ECL3) displayed higher RMSF in WT, while residues 240–245 (TM6 intracellular) and 287 (TM7 extracellular) showed the opposite trend. Within the transmembrane bundle, TM6 and TM7 exhibited significantly larger fluctuations in M39^1.34^R Rho.

#### 3.9.3 Chemical stability

To evaluate the chemical stability of the LYR imine group regarding hydroxylamine accessibility, channels connecting the chromophore binding site with its outer region were identified using Caver software (50 MD structures, 0.75 Å probe radius).

Three main channels with ≥40% occurrence frequency were identified in WT Rho (Figure 9D): Channel A connects the extracellular membrane region to the upper LYR site via TM1/TM7 extracellular ends and ECL2. Channel B bifurcates into two entrances (B1 above TM7 and B2 above TM4) both accessing the upper LYR region through N-terminus, ECL2, ECL3, and TM7. Channel C also bifurcates, with C1 connecting through the membrane region via TM5/TM6 central portions and the β-ionone ring, while C2 connects through TM5/TM6 extracellular ends.

M39^1.34^R Rho preserves channels A, B1, and B2, though with reduced occurrence frequency for A and B1 (Supplementary Table S5). Notably, channel C2 closes while a new channel D emerges, connecting the extracellular region to upper LYR through TM6/TM7 extracellular ends. Channel D exhibits superior properties compared to WT’s C2, with better accessibility from bulk solvent rather than the membrane interface where hydroxylamine diffusion would be limited. Furthermore, the Arg introduced by the M39^1.34^R mutation provides an additional positive charge near channel A that could facilitate hydroxylamine recruitment through electrostatic interactions.

Overall, both variants should display comparable chemical stability, though M39^1.34^R may show increased hydroxylamine reactivity due to the superior accessibility of its differential channel D from bulk solvent, and the enhanced capacity of channel A to attract hydroxylamine molecules through the additional arginine residue.

#### 3.9.4 Screening of prospective cavities

CavityPlus web server identified 11 cavities in both WT and M39^1.34^R Rho, including the orthosteric and G-protein binding sites. To determine potential geraniol binding sites, cavity druggability and average dissociation constants (*K*_d_) were evaluated. Four cavities were excluded due to either superficial location with poor binding properties (3 cavities) or substantial N-terminus involvement with high conformational flexibility (1 cavity).

**Figure 10:**
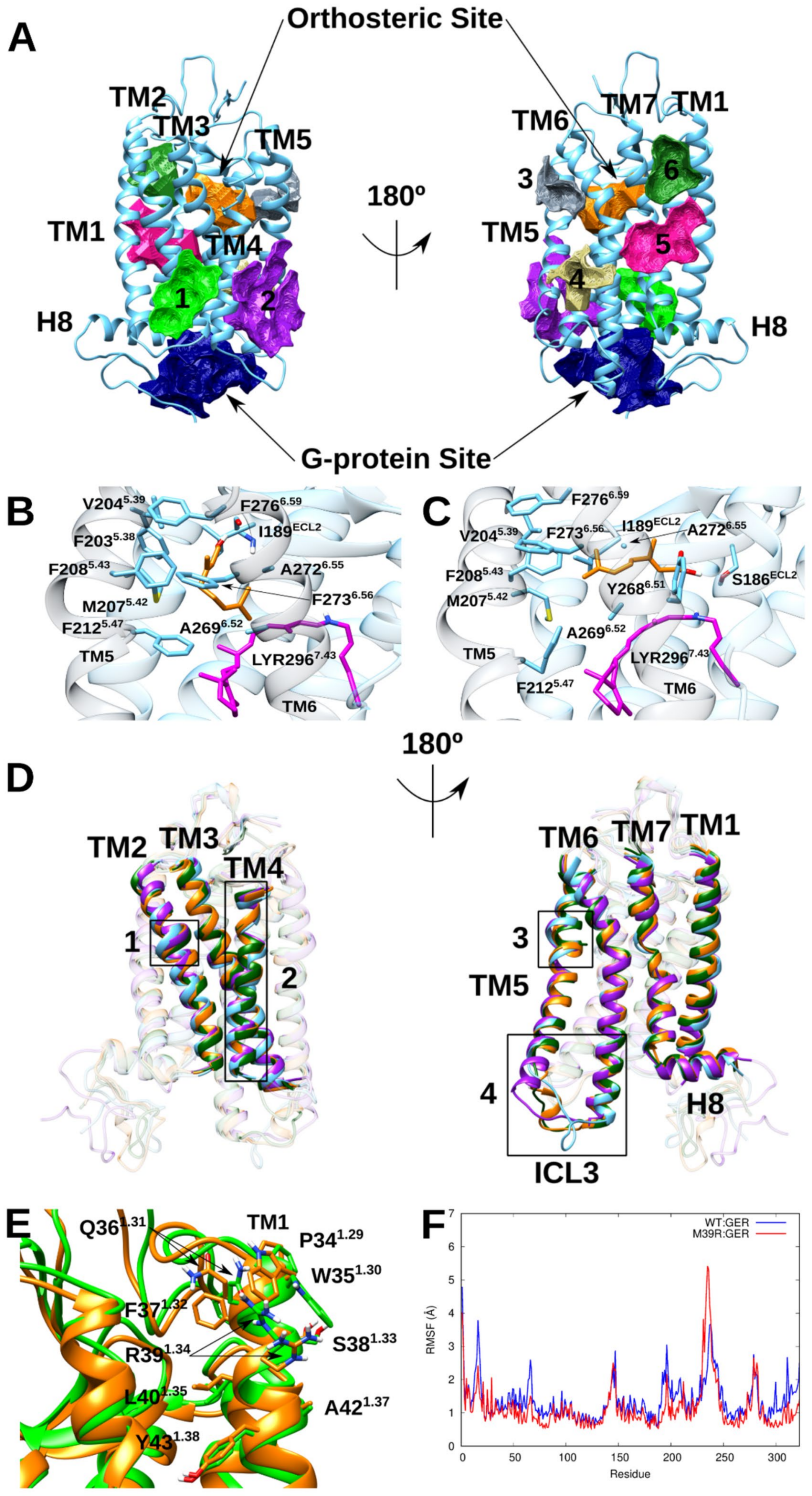
Prospective Rho allosteric cavities to host geraniol together with its possible binding models and structural changes induced by its presence. **(A)** Prospective Rho allosteric cavities, which include both orthosteric and G-protein sites (in orange and navy blue, respectively) and 6 more superficial cavities (cavities 1–6 in green, purple, slate gray, khaki, magenta, and forest green, respectively). **(B,C)** Geraniol binding modes inside cavity 3. In pose 1 **(B)**, geraniol is coiled over the LYR β-ionone ring forming a hydrogen bond with the oxygen backbone atom of Ile189^ECL2^. In contrast, in pose 2 **(C)**, geraniol is totally stretched out over the LYR unsaturated part establishing hydrogen bonds with side chains of Ser186^ECL2^ and Tyr268^6.51^. Both geraniol poses block channel C, while only its pose 2 obstructs channels B and D. For the sake of better spatial orientation, TM5 and TM6 have been highlighted in slate gray. **(D)** Structural overlap of representative structures of WT Rho (in cyan), WT binary complex (in purple), and M39^1.34^R binary complexes (main and minority clusters in orange and dark green, respectively) highlighting main structural changes induced by geraniol presence: (1) Presence of structural defects present on TM2; (2) Increase of TM4 helical content at the membrane region; (3) Presence of structural defects present on TM5; (4) Helical folding of ICL3 region prior to TM6 and of TM5 disordered region located at its intracellular end. **(E)** Environment around the mutant Arg for M39^1.34^R and its corresponding binary complex (in green and orange, respectively). Geraniol presence avoids characteristic stress point of M39^1.34^R mutation disrupting simultaneous interaction between Trp35^1.30^, Gln36^1.31,^ and Arg39^1.34^. **(F)** Per-residue RMSF for WT and M39^1.34^R Rho binary complexes with geraniol.

Among the remaining seven feasible cavities (Figure 10A), the orthosteric and G-protein sites exhibited superior binding and druggability features (Supplementary Table S6), consistent with their biological roles in chromophore and G-protein binding, respectively. The other cavities showed more superficial characteristics with reduced binding potential. Notably, cavity 6 (located between TM1 and TM7 extracellular ends) was the only cavity showing significant differences between variants. The introduction of the positively-charged Arg at position 39^1.34^ impairs this cavity’s hydrophobic character, reducing its ligand-hosting capability in M39^1.34^R Rho.

#### 3.9.5 Geraniol binding mode

To identify stable geraniol binding sites, docking calculations were performed for all prospective cavities in both WT and M39^1.34^R Rho (excluding the LYR-occupied orthosteric site), followed by MD simulations to assess binding stability and conformational effects.

MD simulations revealed that geraniol binds stably exclusively to cavity 3 in both Rho variants, with the compound diffusing into the membrane when initially placed in other cavities. Within cavity 3, geraniol positions itself above the 11CR fragment of LYR, exhibiting considerable conformational flexibility.

Cluster analysis (1.5 Å RMSD threshold, >10% population) identified two distinct binding poses (Figures 10B,C). In pose 1, geraniol adopts a coiled conformation over the β-ionone ring, establishing hydrophobic interactions with TM5/TM6 extracellular ends while its hydroxyl group forms a hydrogen bond with Ile189 backbone (ECL2), associated with lower thermal isomerization rates and higher thermal stability of rhodopsins compared to cone pigments (Kuwayama et al., 2002). In pose 2, geraniol extends along the LYR unsaturated region, maintaining hydrophobic interactions with TM5/TM6 while the hydroxyl group forms weak and transient hydrogen bonds with Ser186 (ECL2) and Tyr268^6.51^. Both poses effectively seal the cavity 3 entrance.

WT Rho accommodates both poses with pose 1 predominating (60.3% vs. 39.7%), whereas M39^1.34^R Rho exclusively adopts pose 2 (97.0%). Notably, geraniol indirectly affects Trp265^6.48^ by causing 11CR reorganization, bringing it closer to this residue and rationalizing the observed fluorescence shift (Supplementary Figure S1). Finally, regarding the described pose 2, it could be argued that geraniol may cause a change in the LYR absorption maximum, since geraniol hydroxyl group is located close to the retinal Schiff base region. However, despite geraniol hydroxyl group points to that region, this does not induce any significant spectral shift since no hydrogen bonds are formed with the retinal Schiff base and moreover, a considerable distance exists between this hydroxyl group and that base (average distance between the oxygen atom of geraniol hydroxyl group and the nitrogen atom of retinal Schiff base is 6.154 Å and 8.480 Å for structures in which geraniol exhibits pose 2 of MD simulations of stable WT Rho-geraniol and M39^1.34^R Rho-geraniol complexes, respectively).

#### 3.9.6 Geraniol impact on structural and chemical features

Geraniol binding to cavity 3 prevents the characteristic stress point formation in M39^1.34^R Rho by blocking Trp35^1.30^ and Gln36^1.31^ reorganization toward Arg39^1.34^, which remains oriented toward bulk solvent (Figure 10E). This results in highly similar conformations between WT and M39^1.34^R Rho-geraniol complexes (Figure 10D).

Structurally, geraniol reverses most M39^1.34^R-induced changes: TM2 and TM5 defects persist, TM4 maintains its helical content without rotation, and both variants gain helical structure at TM4’s membrane region and extracellular end. Secondary structure analysis reveals increased α-helical content for both complexes, more pronounced in WT (2.09% vs. 1.45% increase; Supplementary Tables S7–S9). Geraniol induces variant-specific folding: massive TM5 stabilization in WT Rho (>15% α-helix increase) versus significant TM7 folding in M39^1.34^R (~5% α-helix increase).

Thermal stability shows variant-dependent effects. Geraniol dampens fluctuations in M39^1.34^R Rho (RMSF: 1.070 Å vs. 1.324 Å with and without geraniol, respectively) while amplifying them in WT (1.306 Å vs. 1.223 Å). The differential binding poses explain these patterns: WT accommodates both poses with frequent interchange causing LYR reorganization and increased fluctuations in TM7 intracellular end and H8 along with their associated regions, while M39^1.34^R exclusively maintains pose 2 with reduced dynamics (Figure 10F and Supplementary Table S10).

Regarding chemical stability, geraniol blocks channel C in both variants while differentially affecting other channels (Supplementary Table S11). In WT Rho, channel A is severely impaired with channel B remaining functional. Conversely, M39^1.34^R shows minimal channel A impairment but near-complete blockade of channels B and D. These differences correlate with binding pose preferences: pose 1 (predominant in WT) minimally affects channels B/D, while pose 2 (exclusive in M39^1.34^R) obstructs them through the geraniol hydroxyl group positioning.

Overall, geraniol stabilizes both variants through increased transmembrane helical content and channel restriction. The stabilization is more pronounced for M39^1.34^R Rho due to prevention of mutation-induced defects and enhanced channel blockade, though WT Rho-geraniol remains the most thermally stable complex due to greater helical content gains in TM4/TM5.

## 4 Discussion

This study demonstrates the effects of geraniol, a natural monoterpene alcohol, on the stability of both native Rho and the disease-associated M39^1.34^R mutant. Our results demonstrate that geraniol provides protective effects across multiple assay conditions and provides new insights into pharmacological chaperone strategies for certain Rho mutations associated with RP (Tao and Conn, 2018; Chen et al., 2018).

The thermal stability experiments established geraniol’s protective capacity against heat-induced denaturation of native Rho. This stabilization was reproducible and statistically significant across independent experiments. Chemical stability assays showed similar trends, though the effects were less pronounced, likely due to the inherently high resistance of native Rho to hydroxylamine attack. Importantly, chromophore regeneration kinetics remained unchanged, indicating that geraniol stabilizes the overall protein conformation without compromising the conformational changes required for chromophore binding dynamics.

An unexpected finding emerged from the Meta II experiments. Addition of geraniol to photoactivated Rho induced immediate and substantial fluorescence quenching of Trp265^6.48^, followed by progressive signal decay. This biphasic response suggests both rapid interaction and slower conformational adjustments. During Rho activation, TM6, transmembrane helix in which Trp265^6.48^ resides, undergoes major conformational changes (Altenbach et al., 2008; Farrens et al., 1996) that together with retinal reorganization to adopt its all-trans configuration, potentially creating binding sites absent in the dark state. The partial fluorescence recovery upon protein denaturation confirms that this effect depends on the intact Meta II structure. This observation might indicate that geraniol’s effects extend beyond simple membrane partitioning to include state-dependent protein interactions. However, it should be noted that the measurements extend over more than 100 min, a timescale over which formation of later photointermediates such as Meta III cannot be excluded as a contributing factor to the slower fluorescence decline. Whether geraniol modulates the Meta I/Meta II equilibrium in a pH-dependent manner remains an open question that warrants future investigation. We acknowledge that direct Meta II decay measurements for M39R under comparable experimental conditions were not performed in the present study, representing a limitation that should be addressed in future work to fully establish the relationship between geraniol’s stabilizing effects and the altered phototransduction kinetics previously reported for this mutant.

The M39^1.34^R mutation, associated with sector RP in patients, caused severe thermal instability in our biochemical assays. The dramatic stability deficit can be rationalized by considering the structural consequences of introducing a charged arginine residue in TM1. Methionine at position 39^1.34^ normally participates in hydrophobic interactions that stabilize helix positioning within the transmembrane bundle (Ramon et al., 2014; Park et al., 2008). Its replacement with arginine not only eliminates these favorable contacts but introduces unfavorable electrostatic interactions in the membrane environment. The mutation also increased susceptibility to hydroxylamine, indicating that structural perturbations extend to the chromophore binding region, making the Schiff base more accessible to nucleophilic attack.

Geraniol treatment provided measurable but limited rescue of M39^1.34^R stability defects. While the improvements were statistically significant and proportionally greater than those observed for WT protein, the absolute stability of treated M39^1.34^R remained severely compromised. This enhanced relative benefit for the mutant suggests that geraniol’s stabilizing mechanisms become more valuable when normal structural integrity is disrupted. However, the inability to restore near-normal stability indicates that geraniol alone cannot fully compensate for the structural disruption caused by this severe mutation.

The possibility of direct protein interaction is further supported by previous structural studies showing that amphiphilic molecules with hydrophobic tails and polar headgroups, such as octyl glucoside, can occupy defined surface-accessible cavities in opsin (Park et al., 2013), consistent with a specific binding component beyond simple membrane partitioning.

Here, the mechanism of geraniol action likely involves multiple components operating at different levels. As a lipophilic molecule with a branched hydrocarbon chain and hydroxyl group, geraniol would be expected to partition into membrane bilayers with the alcohol group oriented toward the membrane-water interface. This positioning could influence protein-lipid interactions, potentially stabilizing the transmembrane helices through favorable van der Waals contacts or by modulating local membrane properties such as fluidity or thickness (Soubias and Gawrisch, 2012). The general stabilization observed across different stress conditions supports this membrane-mediated mechanism. The Meta II fluorescence data revealed an additional component involving interaction with the activated state, though whether this represents direct binding or allosteric modulation remains to be determined.

It is worth considering how geraniol’s effects compare to other proposed pharmacological chaperones for Rho. Compounds such as 9-*cis*-retinal have shown ability to rescue certain Rho mutants, primarily through chromophore replacement or modification (Herrera-Hernández et al., 2017). Geraniol appears to operate through a distinct mechanism, providing stabilization without affecting chromophore binding or regeneration. This complementary mode of action suggests potential for combination approaches where multiple compounds targeting different aspects of protein stability could provide additive or synergistic benefits.

Several considerations warrant attention when interpreting these results. First, our experiments utilized purified Rho in DDM micelles, which may not fully recapitulate the complex lipid environment of ROS disc membranes. The native membrane contains specific lipids including docosahexaenoic acid-containing phospholipids that influence Rho stability and function (Niu et al., 2004). It is also worth noting that, despite the theoretical advantage of retaining endogenous lipids, ROS-derived rhodopsin solubilized in DDM does not necessarily outperform recombinant WT in thermal stability assays, as the detergent extraction process itself disrupts the native membrane environment and may introduce sample heterogeneity compared to the more controlled immunoaffinity purification from COS-1 cells. Secondly, we focused exclusively on biochemical stability without examining cellular aspects such as protein trafficking from the endoplasmic reticulum, aggregation in the biosynthetic pathway, or cytotoxicity from misfolded protein accumulation, all of which contribute to disease pathogenesis. Third, geraniol was used at a fixed concentration of 50 μM, selected based on preliminary spectroscopic optimization. While this concentration produced consistent and statistically significant effects across all assays, dose–response characterization and determination of apparent binding affinity remain important goals for future studies to fully establish the pharmacological relevance of the observed stabilization.

The stabilization achieved with geraniol has important implications for therapeutic development. The results demonstrate that small molecules can modulate Rho stability through mechanisms distinct from chromophore replacement. This supports the feasibility of pharmacological chaperone approaches, particularly for mutations with varying degrees of stability defects. Class II mutations causing misfolding but retaining some trafficking capability might be especially suitable candidates. The observation that geraniol provides proportionally greater benefit to destabilized protein suggests a therapeutic window where mutants with intermediate stability defects might achieve more meaningful rescue.

The Meta II interaction deserves particular attention in future studies. Compounds that modulate this signaling state could have broader implications for visual function beyond simple stabilization. The immediate quenching followed by continued slow changes suggests an interaction that could involve initial binding followed by induced conformational adjustments. Understanding this mechanism could guide development of more selective and potent compounds targeting specific Rho conformations.

Future investigations should address several key questions raised by this work. Cellular models would allow evaluation of whether biochemical stabilization translates to improved trafficking, reduced aggregation, and decreased cell death. Structure–activity relationship studies with geraniol analogs could identify molecular features that enhance stabilization. Combination studies with other chaperones or cellular stress modulators might reveal synergistic approaches.

Computational modeling provides molecular-level insights that complement our experimental findings. MD simulations identified cavity 3 as the primary geraniol binding site on Rho’s surface, consistent with both our fluorescence data showing geraniol-induced changes in Trp265^6.48^ environment and the aforementioned complementary mode of action exhibited by geraniol. The M39^1.34^R mutation creates a stress point at the TM1 extracellular end through simultaneous interactions between Trp35^1.30^, Gln36^1.31^, and Arg39^1.34^, inducing greater thermal fluctuations throughout the TM bundle and explaining the experimentally observed instability. Other rationalizations at molecular level could exist, however, according to our calculations and analysis, this is the most feasible explanation. Mutational analysis targeting Trp35^1.30^ and/or Gln36^1.31^ would help to shed light on our hypothesis. Interestingly, geraniol binding to cavity 3 exerts variant-dependent stabilizing effects (preventing detrimental structural changes in M39^1.34^R while increasing TM4 alpha-helical content). The compound appears to channel thermal fluctuations from TM5, absorbing these perturbations without structural compromise. Of particular interest is that, according to our calculations, geraniol in its pose 1 interacts with Ile189, associated with lower thermal isomerization rates and higher thermal stability of Rho (Kuwayama et al., 2002). This interaction would end up increasing WT Rho thermal stability given that such pose is the main binding mode for this Rho variant. However, additional simulations and experiments are needed to unravel the mechanistic implications of the aforementioned interaction regarding the modulation of thermal stability and chromophore dynamics, which we will try to address in future studies. Additionally, geraniol physically obstructs hydroxylamine access channels, with different binding conformations in WT versus M39^1.34^R complexes accounting for the variant-dependent chemical stability effects observed experimentally. These computational insights provide a structural framework for understanding geraniol’s dual mechanism: membrane-mediated stabilization combined with specific binding that modulates both thermal and chemical stability in a mutation-dependent manner.

In conclusion, this work characterizes geraniol as a pharmacological stabilizer of Rho that provides protection against thermal and chemical denaturation through both membrane-mediated effects and specific protein interactions. The compound demonstrates novel state-dependent effects on Meta II and provides enhanced benefit to the destabilized M39^1.34^R mutant, though absolute rescue remains incomplete. These findings contribute to our understanding of how small molecules can modulate Rho stability and support continued investigation of pharmacological chaperones for Rho-associated retinal disease. Geraniol represents a valuable tool compound for studying Rho stabilization mechanisms. The rescue achieved demonstrates the potential of pharmacological approaches and suggests that combination strategies could achieve clinically meaningful outcomes for patients with Rho mutations.

## Data Availability

The raw data supporting the conclusions of this article will be made available by the authors, without undue reservation.
