# Supplementary material for: Stabilizing effects of geraniol on native and pathogenic M39R rhodopsin variants
Source: Front Neurosci. 2026 Mar 20;20:1799935. doi: 10.3389/fnins.2026.1799935 (PMC13047136; doi:10.3389/fnins.2026.1799935)
Supplement: Supplementary file 1 [file Data_Sheet_1.docx]

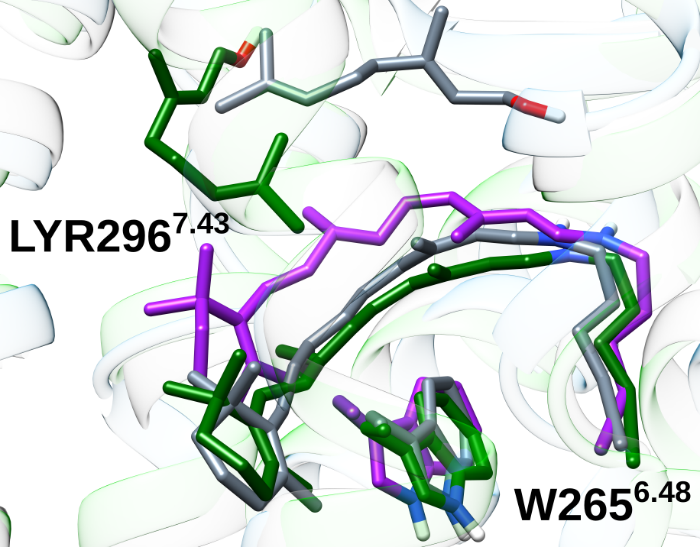


**Figure S1.** Change in the Trp265^6.48^ environment due to LYR reorganizations caused by geraniol presence (geraniol absence in *purple* while poses 1 and 2 of geraniol in *dark green* and *slate gray*, respectively).

**Table S1**. Percentage of the secondary structure content for WT and M39^1.34^R Rho.

| Variant | Parallel β | Anti-parallel β | 3-10 helix | Alpha helix | π helix | Turn | Bend |
| --- | --- | --- | --- | --- | --- | --- | --- |
| WT | 3.532 | 0.322 | 1.804 | 58.630 | 0.002 | 13.059 | 8.331 |
| M39^1.34^R | 3.690 | 0.007 | 2.006 | 58.907 | 0.001 | 11.475 | 10.239 |

**Table S2**. Per-alpha-helix breakdown expressed as a percentage of the secondary structure content for WT Rho.

| Alpha helix | Parallel β | Anti-parallel β | 3-10 helix | Alpha helix | π helix | Turn | Bend |
| --- | --- | --- | --- | --- | --- | --- | --- |
| TM1 | 0.000 | 0.000 | 0.001 | 93.385 | 0.000 | 0.543 | 0.003 |
| TM2 | 0.000 | 0.000 | 0.000 | 90.377 | 0.000 | 3.232 | 0.001 |
| TM3 | 0.000 | 0.000 | 0.002 | 92.013 | 0.000 | 1.840 | 0.005 |
| TM4 | 0.000 | 0.000 | 4.857 | 68.842 | 0.002 | 13.767 | 4.714 |
| TM5 | 0.000 | 0.000 | 0.016 | 65.459 | 0.016 | 8.684 | 6.562 |
| TM6 | 0.000 | 0.000 | 0.000 | 93.826 | 0.000 | 0.830 | 0.080 |
| TM7 | 0.000 | 0.000 | 2.346 | 70.217 | 0.000 | 14.097 | 5.515 |
| H8 | 0.000 | 0.000 | 0.000 | 84.333 | 0.000 | 0.164 | 0.000 |

**Table S3**. Per-alpha-helix breakdown expressed as a percentage of the secondary structure content for M39^1.34^R Rho.

| Alpha helix | Parallel β | Anti-parallel β | 3-10 helix | Alpha helix | π helix | Turn | Bend |
| --- | --- | --- | --- | --- | --- | --- | --- |
| TM1 | 0.000 | 0.000 | 0.002 | 93.288 | 0.000 | 0.628 | 0.014 |
| TM2 | 0.000 | 0.000 | 0.001 | 88.141 | 0.002 | 5.551 | 0.001 |
| TM3 | 0.000 | 0.000 | 0.000 | 92.409 | 0.000 | 1.730 | 0.007 |
| TM4 | 0.000 | 0.000 | 1.328 | 68.900 | 0.000 | 10.257 | 11.770 |
| TM5 | 0.000 | 0.000 | 0.645 | 67.523 | 0.010 | 6.051 | 8.493 |
| TM6 | 0.000 | 0.000 | 0.010 | 93.226 | 0.000 | 1.457 | 0.019 |
| TM7 | 0.000 | 0.000 | 2.619 | 70.641 | 0.000 | 14.011 | 4.863 |
| H8 | 0.000 | 0.000 | 0.000 | 84.328 | 0.000 | 0.219 | 0.000 |

**Table S4**. Per-alpha-helix RMSF (in Å) for WT and M39^1.34^R Rho.

| Variant | TM1 | TM2 | TM3 | TM4 | TM5 | TM6 | TM7 | H8 |
| --- | --- | --- | --- | --- | --- | --- | --- | --- |
| WT | 1.0536 | 0.9265 | 0.8147 | 1.1771 | 1.3457 | 0.9708 | 0.8873 | 0.9706 |
| M39^1.34^R | 1.1095 | 0.9929 | 0.8229 | 1.1879 | 1.3046 | 1.1372 | 1.0307 | 1.1421 |

**Table S5**. Main features of the WT and M39^1.34^R Rho channels that connect the LYR imine group with the GPCR outer region. Freq and Ave BT stand for frequency and average bottleneck radius, respectively. Priority corresponds to an aggregate score that takes into account channel geometric parameters and measures its suitability regarding the entrance/exit of molecules through it. Hyphen symbol indicates that the considered channel is not observed/detected in a reliable way for the corresponding Rho variant.

| Channel/  Entrance | WT | | | | M39^1.34^R | | | |
| --- | --- | --- | --- | --- | --- | --- | --- | --- |
|  | Freq. (%) | Ave BT  (Å) | Ave length  (Å) | Priority | Freq. (%) | Ave BT  (Å) | Ave length  (Å) | Priority |
| A | 74.0 | 0.889 ± 0.105 | 15.183 ± 3.737 | 0.3164 | 56.0 | 0.872 ± 0.111 | 16.032 ± 4.973 | 0.2088 |
| B1 | 80.0 | 1.065 ± 0.157 | 27.301 ± 3.745 | 0.2884 | 60.0 | 0.919 ± 0.145 | 27.521 ± 3.820 | 0.1638 |
| B2 | 42.0 | 0.877 ± 0.100 | 27.108 ± 5.660 | 0.1087 | 42.0 | 1.031 ± 0.190 | 25.851 ± 4.116 | 0.1479 |
| C1 | 52.0 | 0.843 ± 0.072 | 25.383 ± 1.808 | 0.1153 | 46.0 | 0.890 ± 0.127 | 28.273 ± 3.496 | 0.1180 |
| C2 | 40.0 | 0.809 ± 0.044 | 19.949 ± 3.399 | 0.1012 | - | - | - | - |
| D | - | - | - | - | 60.0 | 0.876 ± 0.093 | 18.700 ± 3.840 | 0.2072 |

**Table S6**. Binding and druggable features of the feasible binding hot spots for WT and M39^1.34^R Rho. These features were assessed by average pK_d_, (log(K_d_)) and drugscore, respectively.

| Rho cavity | pK_d_ | | Drugscore | |
| --- | --- | --- | --- | --- |
|  | WT | M39^1.34^R | WT | M39^1.34^R |
| Orthosteric site | 6.87 | 6.87 | 1094 | 1094 |
| G-protein site | 6.94 | 6.94 | 434 | 434 |
| 1 | 4.59 | 4.59 | -939 | -939 |
| 2 | 4.93 | 4.93 | 356 | 356 |
| 3 | 4.92 | 4.92 | -938 | -938 |
| 4 | 5.28 | 5.28 | 185 | 185 |
| 5 | 5.19 | 5.19 | -141 | -141 |
| 6 | 6.31 | 4.58 | 392 | -1279 |

**Table S7**. Percentage of the secondary structure content for WT Rho:Geraniol(3) and M39^1.34^R Rho:Geraniol(3) complexes.

| Variant | Parallel β | Anti-parallel β | 3-10 helix | Alpha helix | π helix | Turn | Bend |
| --- | --- | --- | --- | --- | --- | --- | --- |
| WT | 3.804 | 0.099 | 1.394 | 60.720 | 0.001 | 12.129 | 8.486 |
| M39^1.34^R | 2.546 | 0.494 | 1.744 | 60.359 | 0.014 | 12.371 | 8.081 |

**Table S8**. Per-alpha-helix breakdown expressed as a percentage of the secondary structure content for WT Rho:Geraniol(3) complex.

| Alpha helix | Parallel β | Anti-parallel β | 3-10 helix | Alpha helix | π helix | Turn | Bend |
| --- | --- | --- | --- | --- | --- | --- | --- |
| TM1 | 0.000 | 0.000 | 0.100 | 91.281 | 0.000 | 2.050 | 0.009 |
| TM2 | 0.000 | 0.000 | 0.017 | 87.047 | 0.000 | 6.520 | 0.003 |
| TM3 | 0.000 | 0.000 | 0.001 | 92.518 | 0.000 | 1.526 | 0.005 |
| TM4 | 0.000 | 0.000 | 0.607 | 87.801 | 0.000 | 0.313 | 3.555 |
| TM5 | 0.000 | 0.000 | 0.353 | 82.184 | 0.005 | 10.458 | 0.336 |
| TM6 | 0.000 | 0.000 | 0.172 | 91.685 | 0.000 | 2.654 | 0.169 |
| TM7 | 0.000 | 0.000 | 3.853 | 70.957 | 0.000 | 9.003 | 6.823 |
| H8 | 0.000 | 0.000 | 0.016 | 75.293 | 0.000 | 3.868 | 2.376 |

**Table S9**. Per-alpha-helix breakdown expressed as a percentage of the secondary structure content for M39^1.34^R Rho:Geraniol(3) complex.

| Alpha helix | Parallel β | Anti-parallel β | 3-10 helix | Alpha helix | π helix | Turn | Bend |
| --- | --- | --- | --- | --- | --- | --- | --- |
| TM1 | 0.000 | 0.000 | 0.000 | 93.626 | 0.000 | 0.297 | 0.014 |
| TM2 | 0.000 | 0.000 | 0.005 | 84.213 | 0.143 | 9.151 | 0.004 |
| TM3 | 0.000 | 0.000 | 0.000 | 93.572 | 0.000 | 0.446 | 0.001 |
| TM4 | 0.000 | 0.000 | 0.583 | 88.326 | 0.000 | 0.410 | 2.978 |
| TM5 | 0.000 | 0.002 | 2.217 | 67.959 | 0.004 | 12.093 | 3.443 |
| TM6 | 0.000 | 0.000 | 0.043 | 92.946 | 0.000 | 1.483 | 0.054 |
| TM7 | 0.000 | 0.000 | 0.091 | 75.631 | 0.000 | 8.829 | 7.668 |
| H8 | 0.000 | 0.000 | 0.000 | 84.545 | 0.000 | 0.033 | 0.000 |

**Table S10**. Per-alpha-helix RMSF (in Å) for WT Rho:Geraniol(3) and M39^1.34^R Rho:Geraniol(3) complexes.

| Variant | TM1 | TM2 | TM3 | TM4 | TM5 | TM6 | TM7 | H8 |
| --- | --- | --- | --- | --- | --- | --- | --- | --- |
| WT | 1.3882 | 1.1032 | 0.9229 | 1.2282 | 1.6652 | 1.3593 | 1.1443 | 1.7535 |
| M39^1.34^R | 1.0562 | 0.8723 | 0.8280 | 0.9972 | 1.7047 | 1.0716 | 0.7898 | 0.9845 |

**Table S11**. Main features for the channels of WT Rho:Geraniol(3) and M39^1.34^R Rho:Geraniol(3) complexes that connect the LYR imine group with the GPCR outer region. Freq and Ave BT stand for frequency and average bottleneck radius, respectively. Priority corresponds to an aggregate score that takes into account channel geometric parameters and measures its suitability regarding the entrance/exit of molecules through it. Hyphen symbol indicates that the considered channel is not observed/detected in a reliable way for the corresponding Rho variant.

| Channel/  Entrance | WT | | | | M39^1.34^R | | | |
| --- | --- | --- | --- | --- | --- | --- | --- | --- |
|  | Freq. (%) | Ave BT  (Å) | Ave length  (Å) | Priority | Freq. (%) | Ave BT  (Å) | Ave length  (Å) | Priority |
| A | 48.0 | 0.902 ± 0.108 | 18.142 ± 5.587 | 0.1872 | 46.0 | 0.832 ± 0.072 | 15.255 ± 4.341 | 0.1652 |
| B1 | 74.0 | 1.007 ± 0.197 | 26.345 ± 5.162 | 0.2585 | 16.0 | 0.832 ± 0.063 | 29.679 ± 3.723 | 0.0230 |
| B2 | 36.0 | 0.899 ± 0.119 | 28.625 ± 6.465 | 0.0931 | 14.0 | 0.805 ± 0.052 | 27.997 ± 2.522 | 0.0221 |
| C1 | 34.0 | 0.867 ± 0.132 | 27.015 ± 2.986 | 0.0792 | - | - | - | - |
| C2 | - | - | - | - | - | - | - | - |
| D | - | - | - | - | 22.0 | 0.815 ± 0.106 | 17.933 ± 5.041 | 0.0651 |
